# *Holocephalocotyle monstrosae* n. gen. n. sp. (Monogenea, Monocotylidae) from the olfactory rosette of the rabbit fish, *Chimaera monstrosa* (Holocephali, Chimaeridae) in deep waters off Algeria

**DOI:** 10.1051/parasite/2019060

**Published:** 2019-09-20

**Authors:** Imane Derouiche, Lassad Neifar, Delphine Gey, Jean-Lou Justine, Fadila Tazerouti

**Affiliations:** 1 Université des Sciences et de la Technologie Houari Boumediene (U.S.T.H.B), Faculté des Sciences Biologiques, Département d’Écologie et Environnement, Laboratoire de Biodiversité et Environnement : Interactions et Génomes, Équipe 1 : Parasites : Biodiversité-Bioécologie-Interactions Hôtes-Parasites BP 32, El Alia Bab Ezzouar 16111 Alger Algeria; 2 Laboratoire de Biodiversité Marine et environnement, Faculté des Sciences de Sfax, Université de Sfax BP 1171 3038 Sfax Tunisia; 3 Institut Systématique Évolution Biodiversité (ISYEB), Muséum National d’Histoire Naturelle, CNRS, Sorbonne Université, EPHE, Université des Antilles 57 rue Cuvier, CP 51 75005 Paris France; 4 Service de Systématique Moléculaire, UMS 2700 CNRS, Muséum National d’Histoire Naturelle, CP 26 43 Rue Cuvier 75231 Paris Cedex 05 France

**Keywords:** Chimaeridae, Monocotylidae, *Holocephalocotyle* n. gen., 28S rDNA, Mediterranean

## Abstract

Based on a molecular and morphological study, a new monocotylid genus, *Holocephalocotyle* n. gen. is proposed to accommodate *Holocephalocotyle monstrosae* n. sp., found on the olfactory rosette of the rabbit fish, *Chimaera monstrosa* Linnaeus (Chondrichthyes, Chimaeridae), from the Mediterranean Sea off Algeria. Identification of fish hosts was confirmed by molecular barcoding of the COI gene. A partial 28S rDNA sequence (D1–D2 domain) of *Holocephalocotyle monstrosae* was obtained; it was distinct from all known monocotylid sequences (*p*-distance: 15.5–23%). A phylogenetic tree constructed from available monocotylid sequences showed that *Holocephalocotyle monstrosae* was included, and basal, in a robust group including species of *Merizocotyle, Mycteronastes* and *Empruthotrema*, confirming that the species is a member of the Merizocotylinae. The new genus is unique among the Merizocotylinae in having a distinctive pattern of haptoral loculi with one central, five peripheral and seven “interperipheral loculi” partially inserted between peripheral loculi and a compartmentalised sclerotised male copulatory organ. The diagnosis of the Merizocotylinae is amended to include this new genus. The new genus represents the second monocotylid genus recorded from holocephalans.

## Abstract (fr)

Sur la base d’une étude moléculaire et morphologique, un nouveau genre de Monocotylidae, *Holocephalocotyle* n. gen. est proposé pour accueillir *Holocephalocotyle monstrosae* n. sp., trouvé sur la rosette olfactive de la chimère *Chimaera monstrosa* Linnaeus (Chondrichthyes, Chimaeridae) de la mer Méditerranée au large de l’Algérie. L’identification des hôtes poissons a été confirmée par code-barre moléculaire du gène COI. Une séquence partielle d’ADNr 28S (domaine D1-D2) d’*Holocephalocotyle monstrosae* a été obtenue et était distincte de toutes les séquences de Monocotylidae connues (*p*-distance : 15.5–23 %). Un arbre phylogénétique construit à partir des séquences de Monocotylidae disponibles a montré qu’*Holocephalocotyle monstrosae* était inclus et basal dans un groupe robuste comprenant des espèces de *Merizocotyle*, *Mycteronastes* et *Empruthotrema*, confirmant que l’espèce est membre des Merizocotylinae. Le nouveau genre est unique parmi les Merizocotylinae en ce qu’il présente un motif distinctif de loculi haptoraux avec un loculus central, 5 périphériques et 7 « loculi intra-périphériques » partiellement insérés entre des loculi périphériques, et un organe copulateur mâle sclérifié compartimenté. La diagnose des Merizocotylinae est modifiée pour inclure ce nouveau genre. Le nouveau genre représente le deuxième genre de Monocotylidae répertorié chez les holocéphales.

## Introduction

The Holocephali or chimaerid fishes form a small and enigmatic subclass of Chondrichthyes, hypothetically diverged from a common ancestor with the elasmobranch fishes about 413 Ma ago [27]. Holocephalans are hosts of unique parasite groups including the Chimaericolidae (Monogenea) and the Gyrocotylidea (Cestoda). The rabbit fish, *Chimaera monstrosa*, occurring in the eastern Atlantic Ocean and Mediterranean Sea, is the most studied species among holocephalans [16, 18, 23, 36, 40]. Among metazoan parasites, only two monogeneans were reported from this fish namely, *Chimaericola leptogaster* (Leuckart, 1830) (Chimaericolidae) from the gills and *Calicotyle affinis* Scott, 1911 (Monocotylidae) from the cloaca. However, the olfactory organ has been neglected in previous parasitological examinations; this site could represent, as for numerous elasmobranchs, a potential site for the discovery of new species of monogeneans.

During a collaborative study of the helminth parasites of chondrichthyan hosts of Algeria (western Mediterranean), a species of the Merizocotylinae Johnston and Tiegs, 1922 was recovered from the olfactory chamber of the rabbit fish. In the present study, integrative taxonomy based on molecular and morphological analyses was used to provide a formal description of a new species and the proposal of a new genus for its accommodation. This paper belongs to a series of papers dealing with the morphological and molecular description of monogeneans from off the southern Mediterranean coast [2–7, 22].

## Materials and methods

### Collection of host and parasite material

Between December 2013 and January 2018, a total of 231 specimens of rabbit fish, *Chimaera monstrosa* (7–50 cm in length; 9–1500 g in weight), were collected from five fishing ports in Algeria: 197 from Cherchell (36°36′31″ N, 2°11′50″ E), 15 from Bouharoun (36°37′55″ N, 02°39′35″ E), 10 from El Djamila (36°48′01″ N, 2°54′05″ E), 5 from Annaba (36°54′11″ N, 07°47′03″ E) and 4 from Beni Saf (01°10′23″ W, 35°10′18″ N). The fish were caught during commercial shrimp trawl fishery as bycatch.

All fish caught were dissected shortly after capture and examined for parasites in the laboratory. The olfactory chamber was entirely removed and placed in Petri dishes filled with filtered sea water. The olfactory rosette was scraped out carefully, under a stereomicroscope and monogeneans were removed from the mucus with the help of a dissection needle and aspirated with a pipette. Some living specimens were narcotised with menthol crystal in a 5–10% solution [28], before fixation, to unfold the haptor.

### Molecular analysis

#### Molecular barcoding of host fish

For two individual fish, total genomic DNA was isolated from absolute ethanol fixed olfactory lamellae using a QIAamp DNA Mini Kit, as per the manufacturer’s instructions. The 5′ region of the mitochondrial cytochrome *c* oxidase (COI) subunit I gene was amplified with the primers FishF1 (5′–TCAACCAACCACAAAGACATTGGCAC–3′) and FishR1 (5′–TAGACTTCTGGGTGGCCAAAGAATCA–3′) [39]. PCR reactions were performed in 20 μL, containing 1 ng of DNA, 1× CoralLoad PCR buffer, 3 mM MgCl_2_, 66 μM of each dNTP, 0.15 μM of each primer, and 0.5 units of Taq DNA polymerase (Qiagen). The amplification protocol was 4 min at 94 °C, followed by 40 cycles at 94 °C for 30 s, 48 °C for 40 s, and 72 °C for 50 s, with a final extension at 72 °C for 7 min. PCR products were purified (Ampure XP Kit, Beckman Coulter) and sequenced in both directions on a 3730xl DNA Analyzer 96-capillary sequencer (Applied Biosystems). We used CodonCode Aligner version 3.7.1 software (CodonCode Corporation, Dedham, MA, USA) to edit sequences, which were 651 bp in length, compared them to the GenBank database content with BLAST, and deposited them in GenBank under accession numbers MN397913 and MN397914. Species identification was confirmed with the BOLD identification engine [41].

### Molecular analysis of monogeneans

DNA was extracted using a QiaAmp DNA Micro kit (Qiagen). A 28S rDNA fragment of 884 bp was amplified using the universals primers C1′ (5′–ACCCGCTGAATTTAAGCAT–3′) and D2 (3′–TCCGTGTTTCAAGACGG–5′) [19]. PCR reactions were performed in a final volume of 20 mL, containing: 1 ng of DNA, 16CoralLoad PCR buffer, 3 mM MgCl_2_, 66 mM of each dNTP, 0.15 mM of each primer, and 0.5 units of Taq DNA polymerase (Qiagen). Thermocycles consisted of an initial denaturation step at 94 °C for 1 min, followed by 40 cycles of denaturation at 94 °C for 30 s, annealing at 60 °C, for 30 s, and extension at 72 °C for 1 min. The final extension was conducted at 72 °C for 7 min. PCR products were visualised on a 1.5% agarose gel, purified and directly sequenced in both directions on a 3730xl DNA Analyzer 96-capillary sequencers (Applied Biosystems) at Eurofins Genomics. Sequences were edited and assembled using CodonCode Aligner software (CodonCode Corporation, Dedham, MA, USA), and compared to the GenBank database content with BLAST. Sequences from three individual monogeneans were obtained and were found to be identical; they were deposited in GenBank under accession number MN412655–MN412657.

### Trees of monogeneans and distances

A tree was constructed from our new sequence and 28S sequences of monocotylids (Table 1). We used almost all sequences available in GenBank, with the exception of sequences that were too short, such as those of *Loimosina* sp. (KF908848), *Potamotrygonocotyle tsalickisi* (JN379513), and *Calicotyle affinis* (AF382061), the latter of which did not align well (but five other species of *Calicotyle* were included in the analysis); a sequence of the microbothriid *Leptocotyle minor* was used as the outgroup. The dataset included 32 nucleotide sequences. Both extremities of sequences were trimmed to obtain a clean matrix and thus there were 744 positions in the dataset. After estimating the best model with MEGA7 [26], the tree was inferred using the Maximum Likelihood method based on the General Time Reversible model [32] with gamma distribution and invariant sites (GTR + G + I) in MEGA7 [26], with 200 replications. The neighbour-joining method was also used for comparison in MEGA7, with bootstrap calculated on 2000 replicates. Distances between sequences (*p*-distances) were computed from the same dataset with MEGA7 [26].

**Table 1:** Species of the Monocotylidae used in the molecular analysis.

Parasite species	Host species	Site	Locality	GenBank ID
*Holocephalocotyle monstrosae*	*Chimaera monstrosa*	Olfactory rosette	Algeria, Mediterranean Sea	MN412655–MN412657
*Calicotyle kroyeri*	*Raja radiata*	Cloaca	North Sea	AF279746
*Calicotyle palombi*	*Mustelus mustelus*	Cloaca	Tunisia, Mediterranean Sea	AF279749
*Calicotyle stossichi*	*Mustelus mustelus*	Rectal gland	Tunisia, Mediterranean Sea	AF279751
*Calicotyle urolophi*	*Urolophus cruciatus*	Cloaca	Tasmania, Australia	AF279752
*Calicotyle japonica*	*Squalus mitsukurii*	Cloaca	Pacific	AB485996
*Dictyocotyle coeliaca*	*Raja radiata*	Inner wall of body cavity	North Sea	AF279754
*Empruthotrema dasyatidis*	*Orectolobus maculatus*	Nasal tissue	Heron Island, Australia	AF348345
*Empruthotrema quindecima*	*Taeniura lymma*	Nasal tissue	Heron Island, Australia	AF348346
*Merizocotyle urolophi*	*Urolophus bucculentus*	Nasal tissue	Tasmania, Australia	AF348347
*Merizocotyle australensis*	*Himantura fai*	Nasal tissue	Heron Island, Australia	AF348348
*Merizocotyle sinensis*	unknown	unknown	unknown	FJ514075
*Mycteronastes icopae*	*Rhinobatos typus*	Nasal tissue	Heron Island, Australia	AF348349
*Clemacotyle australis*	*Aetobatus narinari*	Gills	Heron Island, Australia	AF348350
*Dendromonocotyle ardea*	*Pastinachus sephen*	Dorsal skin	Heron Island, Australia	AF348351
*Dendromonocotyle octodiscus*	*Dasyatis americana*	Dorsal skin	Gulf of Mexico, Mexico	AF348352
	*Urobatis jamaicensis*			
*Monocotyle corali*	*Pastinachus sephen*	Gills	Heron Island, Australia	AF348353
*Monocotyle helicophallus*	*Himantura fai*	Gills	Heron Island, Australia	AF348355
*Monocotyle spiremae*	*Himantura fai*	Gills	Heron Island, Australia	AF348354
*Monocotyle multiparous*	*Himantura fai*	Gills	Heron Island, Australia	AF348356
*Decacotyle floridana*	*Aetobatus narinari*	Gills	Heron Island, Australia	AF348357
*Decacotyle tetrakordyle*	*Pastinachus sephen*	Gills	Heron Island, Australia	AF348358
*Decacotyle lymmae*	*Aetobatus narinari*	Gills	Heron Island, Australia	AF348359
*Heterocotyle capricornensis*	*Himantura fai*	Gills	Heron Island, Australia	AF348360
*Neoheterocotyle rhinobatidis*	*Rhinobatos typus*	Gills	Heron Island, Australia	AF348361
*Neoheterocotyle rhinobatis*	*Rhynchobatus djiddensis*	Gills	Heron Island, Australia	AF348362
*Neoheterocotyle rhynchobatis*	*Rhinobatos typus*	Gills	Heron Island, Australia	AF348363
*Troglocephalus rhinobatidis*	*Rhinobatos typus*	Gills	Heron Island, Australia	AF348364
*Electrocotyle whittingtoni*	*Narke capensis*	Gills	South Africa	KT735368
*Potamotrygonocotyle dromedarius*	*Potamotrygon motoro*	Gills	Brazil	JN379518
*Potamotrygonocotyle chisholmae*	*Potamotrygon motoro*	Gills	Brazil	JN379519
*Leptocotyle minor*	*Scyliorhinus canicula*	Skin	North Sea	AF382063

### Morphological analysis

Five specimens were prepared as whole mounts in Malmberg solution (*m*) [29] to study the morphology of anchors and the male copulatory organ. Forty specimens were flattened under slight coverslip pressure and fixed in 70% ethanol and thereafter stained with acetic carmine (*c*) before being dehydrated in a graded ethanol series, cleared in clove oil, and permanently mounted in Canada balsam.

Measurements were obtained using drawing tube and ruler calibrated using a microscopic graduated slide. They represent straight-line distances between extreme points. Measurements are in micrometers, and indicated as means ± standard deviation (if *n* > 30), and between parentheses the range and number (*n*) of structures measured. Because measured lengths may vary depending on how specimens are prepared and the degree to which they are flattened [2, 21], they are given separately for specimens mounted respectively in Malmberg’s medium (*m*) or stained in carmine and mounted in Canada balsam (*c*).

Drawings of the parasite were made using a Leitz microscope equipped with a drawing tube and redrawn afterwards on a computer with Adobe Illustrator. Terminology of the olfactory organ of *Chimaera monstrosa* follows that described by [17]. Haptoral terminology follows that presented and illustrated by [11, 25].

The term uncinuli (singular uncinulus) is used for marginal hooklets after Pariselle & Euzet (1995) [37]. Uncinuli numbering follows that proposed by Kritsky et al. (2017) [25]. We propose the term interperipheral loculi for the loculi partially inserted between peripheral loculi in the haptor. Type specimens of the new taxa were deposited in the Collections of the Muséum National d’Histoire Naturelle (MNHN), Paris.

## Results

### Molecular analysis

#### Molecular identification of fish

The provisional identification of fish species using morphological characteristics was reconfirmed by a DNA barcoding approach. BLAST analysis of the COI sequences of sequences of two host fish specimens with NCBI and BOLD database showed sequence similarity values of, respectively 99.38% and 100% with *Chimaera monstrosa*. The BOLD database [41] includes many sequences with published information for this species and we are thus confident that the identification is valid.

#### Phylogenetic analysis of monogeneans from 28S sequences

In our dataset, the genetic distance between our new sequences and all monocotylid sequences ranged from 15.5% to 23%, with the closest sequence being *Empruthotrema dasyatidis* (15.5%) and the most different sequences being *Dendromonocotyle ardea* (23%); this clearly showed that our species is different from all other sequenced monocotylid species.

**Figure 1:**
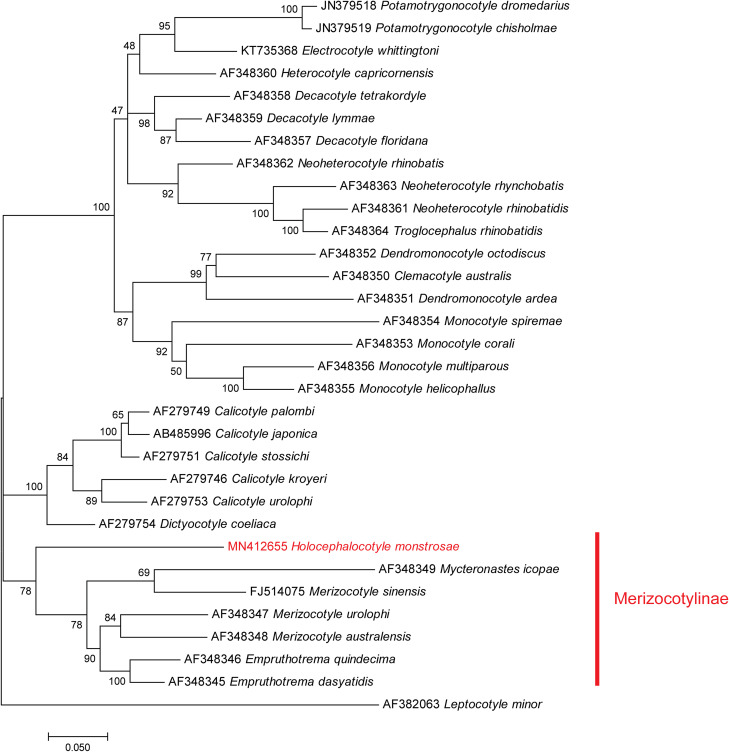
Maximum Likelihood tree of the Monocotylidae based on an analysis of 28S rDNA sequences. Bootstrap percentages with 200 replicates.

A tree built from 32 sequences, with *Leptocotyle minor* (Microbothriidae) as the outgroup and 31 monocotylid sequences including the new sequence from our species (Table 1), provided the following results (Fig. 1). There was a total of 744 positions in the final dataset. The general topology of the tree, constructed with the Maximum Likelihood method, showed general agreement with the classification of species into genera, with most genera monophyletic; however, some nodes showed low support. A branch of the tree included species of *Merizocotyle*, *Mycteronastes* and *Empruthotrema* (three genera which are members of the Merizocotylinae) and our new sequence. An analysis based on the NJ method (not shown) showed a slightly different topology of the tree but the Merizocotylinae clade was also clearly defined. Support for the monophyly of the Merizocotylinae clade was high (78% of trees with ML, bootstrap 78 with NJ), strongly suggesting that our new species is a member of the subfamily Merizocotylinae. Within this clade, the new species is sister-group to a clade including all other species (three members of *Merizocotyle,* one *Mycteronastes* and two members of *Empruthotrema*). Within this clade, members of *Merizocotyle* apparently do not constitute a monophylum, with *Merizocotyle sinensis* being a sister-group of *Mycteronastes icopae*.

### Amended diagnosis of Merizocotylinae Johnston & Tiegs, 1922

With characters of family Monocotylidae Taschenberg, 1879 (*sensu* Chisholm, Wheeler & Beverley-Burton [11]). Haptor with one central loculus (absent or replaced by a central depression in *Mycteronastes)*, one to four interhamular loculi (*sensu* Kritsky et al. [24]) and four to seven peripheral loculi. Interperipheral loculi sometimes present. Marginal loculi usually present. Haptor rarely with three loculi or numerous unevenly distributed loculi. Fourteen marginal uncinuli. Three prominent apertures from glands containing needle-like secretion on each side of anterior end. Intestinal caeca unbranched, not confluent posteriorly. Testes single, ovoid. Male copulatory organ (MCO) sclerotised (except in *Mycteronastes undulatae* and *Mycteronastes caalusi*). Ovary not lobed at blind end. Two vaginae; walls of vaginae not sclerotised. Parasites of fishes of Elasmobranchii and Holocephali.

Type genus: *Merizocotyle* Cerfontaine, 1894.

Additional genera: *Cathariotrema* Johnston and Tiegs, 1922; *Empruthotrema* Johnston and Tiegs, 1922, *Mycteronastes* Kearn & Beverley-Burton, 1990, *Squalotrema* Kearn and Green, 1983; *Thaumatocotyle* Odhner, 1910, *Triloculotrema* Kearn, 1993; and *Holocephalocotyle.*

### *Holocephalocotyle* n. gen.

Zoobank: urn:lsid:zoobank.org:act:80808F18-10D7-4CD2-974C-C6A10DB55F96

With characters of family Monocotylidae (*sensu* Chisholm et al. [11]), and subfamily Merizocotylinae. Haptor with one central, five peripheral and seven interperipheral loculi partially inserted between peripheral loculi. Seven pairs of marginal uncinuli. Male copulatory organ with muscular bulb. Parasites of the nasal tissue of Chimaeridae.

*Etymology*: The generic name was constructed based on the name of the subclass of the host (Holocephali) and the ending *– cotyle*. Gender: feminine.

### *Holocephalocotyle monstrosae* n. sp. (Fig. 2)

Zoobank: urn:lsid:zoobank.org:act:153B8EF4-AA14-48BC-BCA6-1E8DD614E3C4

**Figure 2:**
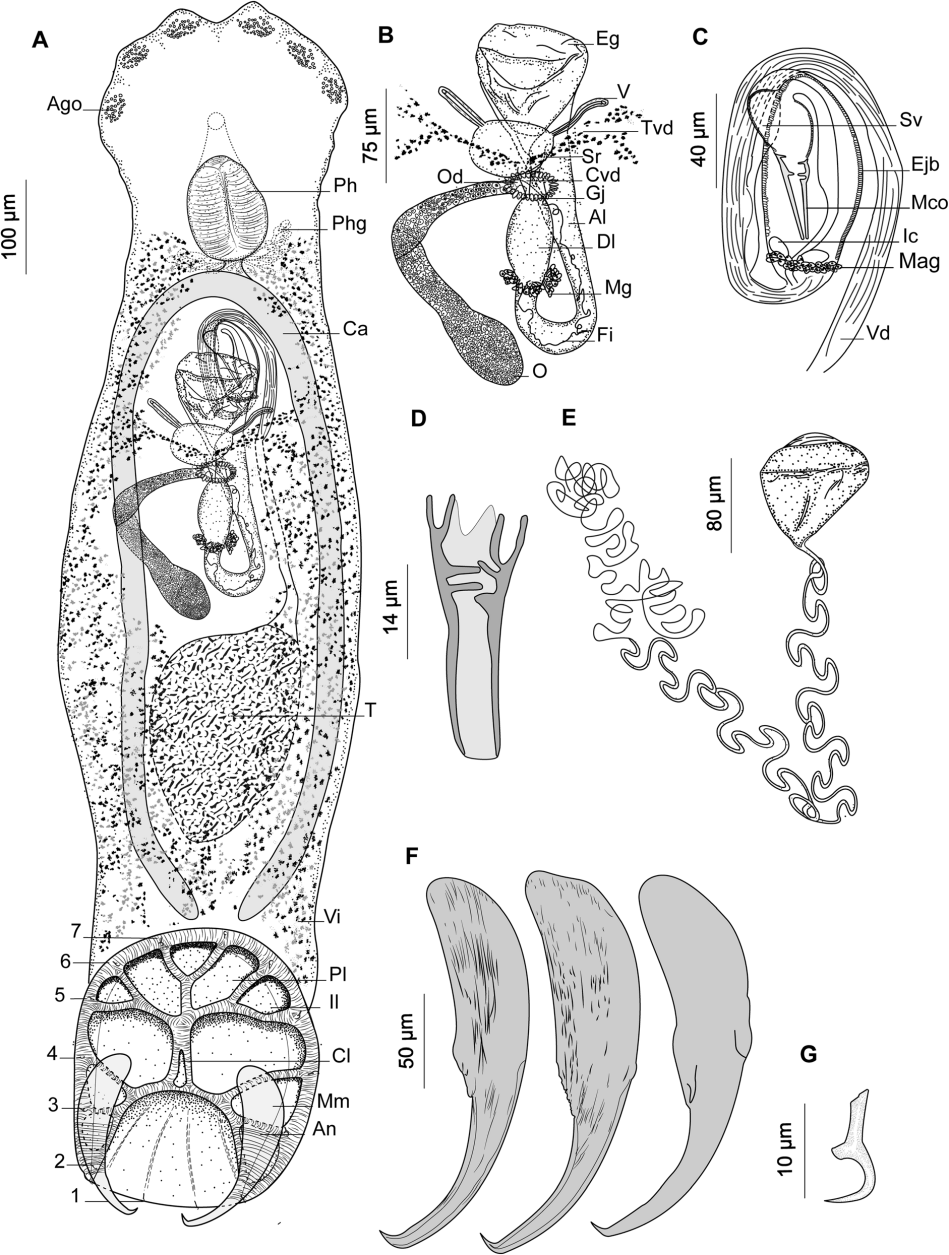
*Holocephalocotyle monstrosae* n. gen. n. sp. from *Chimaera monstrosa*. (A) Holotype, whole body (Ago, anterior gland duct openings; Ph, pharynx; Phg, pharyngeal glands; Ca, caecum; T, testis; Vi, vitellarium; Pl, peripheral loculus; Il, interperipheral loculus; Cl, central loculus; Mm, marginal membrane; An, anchor); (B) holotype, female reproductive system (Eg, egg; V, vaginae; Tvd, transverse vitelline duct; Sr, seminal receptacle; Cvd, common vitelline duct; Od, oviduct; Gj, glandular junction; Al, ascending limb of oötype; Dl, descending limb of oötype; Mg, Mehlis’ glands; Fi, filament; O, ovary); (C) holotype, male reproductive system, (testis not depicted [Sv, seminal vesicle; Ejb, ejaculatory bulb; Mco, male copulatory organ; Ic, internal chamber; Mag, male accessory glands; Vd, vas deferens]); (D) male copulatory organ; (E) egg; (F) anchors, variations according to different specimens; (G) uncinulus; (1–7) uncinuli.

Type host: *Chimaera monstrosa* Linnaeus (Chimaeriformes: Chimaeridae).

Type locality: El Djamila port (36°48′01″ N, 2°54′05″ E), Algeria.

Other locality: Cherchell port (36°36′31″ N, 2°11′50″ E), Algeria.

Site on the host: Olfactory lamellae.

Type material: Holotype (MNHN HEL1105) and 11 paratypes (MNHN HEL1106–HEL1116).

Molecular sequence data: The 884 bp ribosomal DNA sequences covering the D1–D2 domains of the 28S rDNA gene of three specimens were deposited in GenBank under accession numbers MN412655–MN412657. The individual monogenean barcoded as MN412656 was from the individual fish barcoded as MN397913.

Prevalence: 43% (*n* = 231); abundance: 0.49; maximum intensity: 6.

Etymology: The species is named after the Latin specific name of the host, “*monstrosa*”.

#### Description

Based on 40 specimens in carmine (*c*) and five specimens in Malmberg solution (*m*). Body length 1398 ± 368 (1230–1970, *n* = 40) (*c*), 1808 (1350–2400, *n* = 5) (*m*) long, including haptor; maximum width 315 ± 60 (200–480, *n* = 40) (*c*), 422 (321–715, *n* = 5) (*m*) at level of ovary. Haptor longer than broad, 346 (253–402, *n* = 8) (*c*), 420 (350–558, *n* = 5) (*m*) long; 297 (231–334, *n* = 8) (*c*), 375 (436–340, *n* = 5) (*m*) wide. Haptor with one central, five peripherals, and seven interperipheral loculi. Anchor robust (Fig. 2F) 199 ± 23 (147–257, *n* = 51) (*c*), 205 (181–224, *n* = 7) (*m*) long with bilaterally flattened handle and curved blade. Fourteen (seven pairs) marginal uncinuli with straight shaft and sickle (Fig. 2G) 13 ± 1 (11–16, *n* = 52) (*c*), 13 (12–14, *n* = 7) (*m*) long; uncinuli distribution as shown in Figure 2A.

Three openings containing what appears to be needle-like secretion open on each side of ventrolateral margin of anterior end. Anterior glands indistinct. Pharynx ovate 107 ± 27 (93–190, *n* = 40) (*c*), 145 (117–190, *n* = 4) (*m*) long, 92 ± 23 (80–150, *n* = 40) (*c*), 136 (115–180, *n* = 4) (*m*) wide; Pharyngeal glands present; Oesophagus short.

Testis oval, 307 ± 93 (110–480, *n* = 37) (*c*), 345 (337–350, *n* = 2) (*m*) long, 181 ± 64 (100–350, *n* = 37) (*c*), 159 (157–160, *n* = 2) (*m*) wide. Vas deferens arises from left side of testis, runs anteriorly dorsal to transverse vitelline duct. Vas deferens inflates to form seminal vesicle and curves ventral to ejaculatory bulb to right side of the body (Fig. 2C). Seminal vesicle runs posteriorly, narrows and enters posterior part of ejaculatory bulb. Ejaculatory bulb muscular elongate 119 (56–235, *n* = 25) (*c*), 210 (210–210, *n* = 1) (*m*) long, 43 (20–91, *n* = 25) (*c*), 40 (*n* = 1) (*m*) wide, with two distinct internal chambers (Fig. 2B). Male accessory glands lateral on either side of posterior end of the ejaculatory bulb. MCO (Fig. 2D) 45.1 ± 5.2 (35–58, *n* = 34) (*c*), 42 (40–45, *n* = 4) (*m*) long, sclerotised, funnel shaped and distally compartmentalised.

Ovary loops right intestinal caecum dorsoventrally and narrows to form oviduct (Fig. 2B). Oviduct receives duct from vagina and common vitelline ducts in glandular junction. Ovovitelline duct runs posteriorly to join oötype. Mehlis’ glands at base of descending limb of oötype. Oötype forms short descending and ascending limbs and widens at anterior end. Vaginal pores open at level posterior to common genital pore. Two vaginal canals running parallel to transverse vitelline duct to connect seminal receptacle.

Vitellarium extends from level of posterior part of pharynx to posterior part of body proper. Transverse vitelline duct at level of anterior portion of ovary. Egg tetrahedral with long filament (Fig. 2E) 80 (60–100, *n* = 20) (*c*), 95 (90–100, *n* = 2) (*m*) long, 63.5 (44–90, *n* = 20) (*c*), 75 (70–80, *n* = 2) (*m*) wide.

#### Differential diagnosis

*Holocephalocotyle monstrosae* n. gen. n. sp. can be differentiated from the other species of the 29 genera within the Monocotylidae on the basis of the arrangement of the haptoral loculi. The haptoral loculi have a distinctive pattern in which the interperipheral loculi are partially inserted between peripheral loculi (Fig. 2). This feature cannot be accommodated into any of the established genera in the Monocotylidae.

According to the presence of three distinct apertures on each side of the anterior margin into which open ducts from the lateral glands containing a needle-like secretion together with oötype with a descending and ascending limb, we place the parasite of *Chimaera monstrosa* among the Merizocotylinae [13]. Within this subfamily this parasite has a very unique haptor, which allows it to be considered a new species, type of a new genus. This feature was included in the diagnosis of the subfamily to accommodate this new species in a new genus. The amendment of the subfamily was supported by the phylogenetic tree. With the erection of *Holocephalocotyle* n. gen., the number of genera belonging to the Monocotylidae increases to 30.

## Discussion

Interperipheral loculi of *Holocephalocotyle monstrosae* may represent marginal loculi that have been shifted between the peripheral loculi. In the absence of a phylogenetic analysis conducted using morphological characters, homology of these characters cannot be assumed.

Based on a phylogeny generated from morphological data, Chisholm et al. [11] recognised five genera among the Merizocotylinae Johnston & Tiegs, 1922: *Merizocotyle* Cerfontaine, 1894; *Cathariotrema* Johnston & Tiegs, 1922; *Empruthotrema* Johnston & Tiegs, 1922; *Squalotrema* Kearn & Green, 1983 and *Triloculotrema* Kearn, 1993. This analysis failed to demonstrate synapomorphies for *Merizocotyle* (*sensu* Cerfontaine), *Mycteronastes* Kearn & Beverley-Burton, 1990 and *Thaumatocotyle* Odhner, 1910 so the three genera were synonymised with the senior *Merizocotyle*.

Chisholm et al. [10] provided a phylogenetic analysis of the Monocotylidae inferred from 28S rDNA and concluded that *Merizocotyle* was paraphyletic and suggested that *Mycteronastes* and *Thaumatocotyle* should perhaps be resurrected as valid genera. Neifar et al. [35], de Buron & Euzet [15], and Marie & Justine [30] recognised *Thaumatocotyle* as a valid genus. Kritsky et al. [24] resurrected *Mycteronastes* and amended the diagnosis of this genus.

The resurrection of *Thaumatocotyle* and *Mycteronastes* seems to be supported by host-parasite phylogenetic relationships. Species of *Merizocotyle* (*sensu* Cerfontaine) are usually parasites of Rajidae, members of *Thaumatocotyle* are all parasites of Dasyatidae, and members of *Mycteronastes* are parasites of Rhinopristiformes (as proposed by [1, 31]). *Merizocotyle urolophi*, a parasite of *Urolophus paucimaculatus* (Urolophidae), may represent a separate genus, as suggested by Chisholm & Whittington [14], but closely related to *Thaumatocotyle* as shown by the current phylogeny.

The position of *Merizocotyle sinensis* is enigmatic. This species is a parasite of *Platyrhina sinensis*, a Rhinobatidae and not a Dasyatidae as indicated by Chisholm & Whittington [13]. It has a haptor with seven peripheral loculi like the other species of *Merizocotyle* (*sensu* Cerfontaine), but our phylogenetic analysis, based on a partial 28S rDNA unpublished sequence deposited in GenBank, placed this species as a sister-group to *Mycteronastes icopae*, and not with the two other species of *Merizocotyle* (Fig. 1); if *Merizocotyle sinensis* is kept in *Merizocotyle*, *Merizocotyle* is not monophyletic based on our analysis*. Merizocotyle sinensis* might represent a distinct genus as suggested by Kritsky et al. [24].

Our distance analysis also showed that the sequence of the partial 28S rDNA of *Holocephalocotyle monstrosae* is distinct from all other monocotylids by a significant distance (15.5–23%). Our phylogenetic analysis based on partial 28S sequences confirmed that it is a member of the Merizocotylinae, and that it was the sister-group of the other species of Merizocotylinae included in the analysis; however, available sequences of Merizocotylinae included only members of three genera, *Mycteronastes, Merizocotyle* and *Empruthotrema*, which constitute only a subset of the seven genera previously known in this subfamily.

The ejaculatory bulb of *Holocephalocotyle monstrosae* has two internal chambers. These chambers were also reported from the Calicotylinae [9], Heterocotylinae [34], Euzetiinae [14, 38] and numerous merizocotylines (e.g., *Cathariotrema selachii* [33] and *Mycteronastes caalusi* Kritsky, 2017, in which they were described as prostatic reservoirs). This character is not always visible and should be checked for other monocotylids.

The microhabitat seems to have an important impact on the evolution of monocotylids [12]. *Holocephalocotyle monstrosa* and the other Merizocotylinae are mainly parasites of the nasal cavity. Locations of merizocotylines in other microhabitats than the nasal cavity have been widely discussed by Chisholm & Whittington [13] and might be erroneous. Species of Calicotylinae have been described from the cloaca, rectum, rectal gland, spiral valve, oviduct and inner wall of the body cavity [9]. Species of the other subfamilies, Dasybatotreminae, Decacotylinae, Heterocotylinae, Monocotylinae and Euzetiinae, were reported from different microhabitats such as the skin and gills.

**Figure 3:**
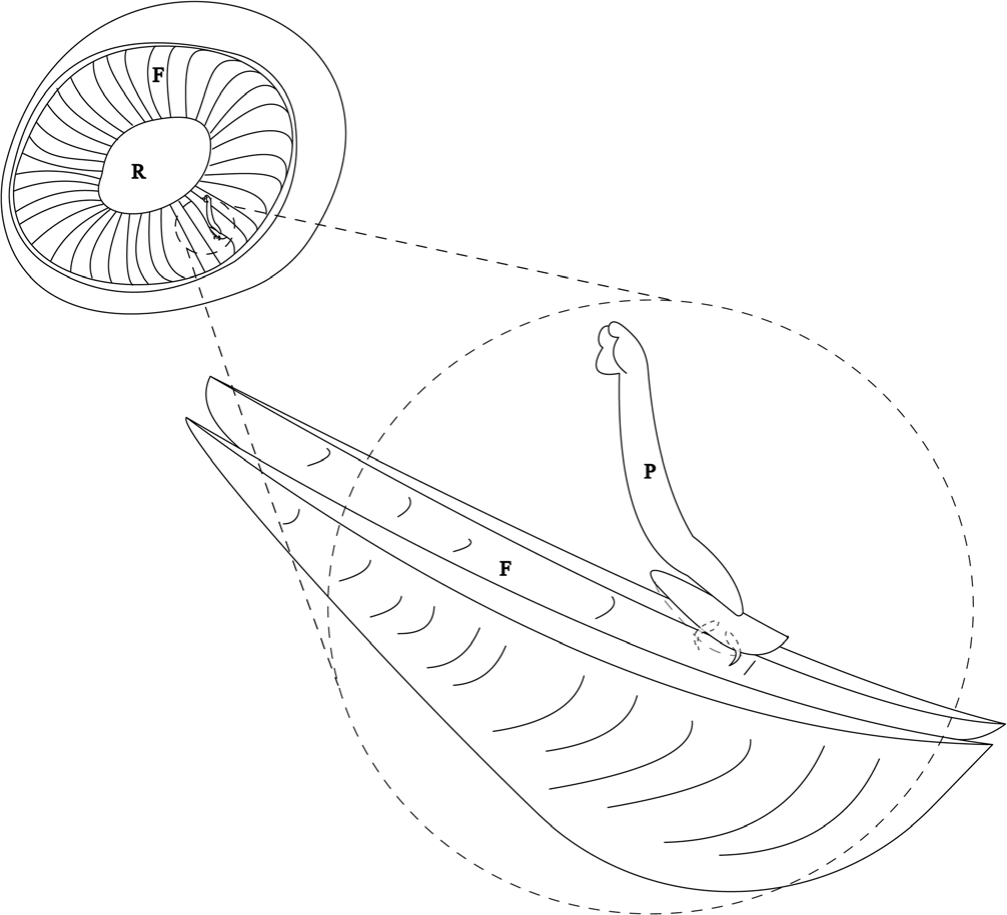
Mode of attachment of *Holocephalocotyle monstrosae* n. gen. n. sp. in the olfactory fold. F, Fold; R, Raphe; P, Parasite.

The haptor of *Holocephalocotyle monstrosae* attaches to the free edge of the primary nasal fold by folding longitudinally and embedding the blade of the anchors into secondary folds (Fig. 3). *Holocephalocotyle monstrosae* does not attach to the glass of the Petri dish by its haptor but rather the haptor remains folded with the blades of the anchors protruding. The same mode of attachment was observed for *Monocotyle corali* [8], a gill parasite of *Pastinachus sephen*. Presence of the loculi and attachment by folding ensure effective anchoring for *H. monstrosae*. The olfactory rosette is exposed to a strong water current. Howard [20] estimated that 20–50% of inspired ﬂow of respiratory water passes through secondary folds of the olfactory rosette and then passes into the gills. It remains to be studied how the parasitism by *Holocephalocotyle monstrosae* may affect the respiratory efficiency of the host.
